# Effectual Use of the Sponge Tent in Sterility

**Published:** 1860-12

**Authors:** 


*Effectual Use of the Sponge Tent in Sterility.*—M. Pieffer mentions, in *L'Union Medicale* of the 28th ultimo, that Prof. Stalz, of Strasbourg, succeeded in removing sterility in the case of a healthy childless couple, who had been married four years. On examination, the cervix was found extremely narrow and very rigid. The use of tents of prepared sponge for a month or six weeks, with an occasional warm bath of an hour's duration, was advised; and the lady became pregnant two months after beginning the treatment. She was eventually delivered of a healthy boy. This procedure seems to M. Pieffer preferable to the division of the cervix, as advised by Dr. Simpson, especially where the patients object to the use of the knife.—*London Lancet.*

